# Diagnostic value of the creatine kinase-MB/creatine kinase and neutrophil/lymphocyte ratios in acute myocardial infarction

**DOI:** 10.1186/s13019-024-02740-9

**Published:** 2024-04-16

**Authors:** Yin Liu, Xiao-Lei Tang, Yong Ni, Li-Zhong Duan, Feng-Jun Jing

**Affiliations:** 1 Clinical laboratory, Traditional Chinese Medicine Hospital of Wuhu, Wuhu, 241000 China; 2 Science and technology department, The Second Affiliated Hospital of Wannan Medical College, No.10, Kangfu Road, Jinghu district, Wuhu City, 241000 Anhui Province China

**Keywords:** CK-MB/CK ratio, neutrophil/lymphocyte ratio, Acute myocardial infarction

## Abstract

**Objective:**

To investigate the clinical significance of the creatine kinase (CK)-MB/total CK ratio, neutrophil/lymphocyte ratio (NLR) and red blood cell distribution width in acute myocardial infarction (AMI).

**Methods:**

A retrospective analysis was conducted of 196 AMI cases from our hospital’s cardiology department; healthy people were selected over the same period as the control. The two groups’ test indexes were compared through multivariate logistic regression analysis to screen for AMI risk factors; the receiver operating characteristic (ROC) curve was used to evaluate their AMI predictive values.

**Results:**

The serum CK, CK-MB, CK index, neutrophils and NLR values in the AMI group were significantly higher compared with those in the control group (*p* < 0.05); however, the levels of serum lymphocytes were significantly lower compared with those in the control group (*p* < 0.05). Multivariate logistic regression analysis showed that elevated CK-MB and NLR levels were risk factors for AMI (*p* < 0.05). The ROC curve showed that the area under the curve of the NLR and CK levels were 0.917 and 0.594, respectively.

**Conclusion:**

The CK index and NLR have a clinical predicting value for AMI and could be used as a clinical auxiliary diagnostic index for the assessment of patients with AMI.

## Introduction

Acute myocardial infarction (AMI) is a severe type of ischemic heart disease. Globally, approximately 9 and 182 million deaths and disabilities, respectively, were caused by AMI in 2019 [1–3]. Creatine kinase-MB (CK-MB) is a clinically recognised biomarker for detecting myocardial injury. It is mainly present in myocardial tissue; when myocardial cells experience ischemia or necrosis, CK-MB is released into the blood, resulting in an elevated CK-MB index in the serum. Inflammation plays an important role in the occurrence and progression of AMI and the neutrophil (NEUT)/lymphocyte (LYM) ratio (NLR) is a marker of the body’s inflammatory state. The NLR is a simple and low-cost measure that is associated with the development and prognosis of various diseases [4–6]. Therefore, this study aimed to explore the diagnostic value of the CK index and NLR in patients with AMI and further investigate their predictive values for disease progression.

## Materials and methods

### Study participants

The calculated sample size was at least 190 cases based on the following sample size calculation formula for case-control studies:

$$N\, = \,{Z^2}*{\sigma ^2}/{d^2}$$

where *Z* is the confidence interval, *N* is the sample size, *d* is the sampling error range and σ is the standard deviation (usually taken as 0.5). Therefore, a total of 196 patients with AMI (110 men and 86 women, aged 33–91, average = 75.2 ± 12.4 years) diagnosed in our hospital between December 2019 and July 2022 were selected via random sampling as the AMI group. All selected patients met the diagnostic criteria for AMI released by the Chinese Society of Cardiology. Additionally, 144 healthy individuals (83 men and 61 women, aged 35–89, average = 69.3 ± 11.9 years) who underwent physical examinations during the same period were selected as the control group.

The exclusion criteria were as follows: patients with (1) severe cardiovascular and cerebrovascular diseases of other types; (2) severe liver and kidney dysfunction or undergoing haemodialysis; (3) recent major surgery or serious traumatic history; and (4) severe immune system diseases or recent use of immunosuppressants, malignant tumours or undergoing radiotherapy or chemotherapy, among others.

Outliers in the data were identified and removed using the interquartile range (IQR) method. The IQR is the difference between the 75th and 25th percentiles of the data. Any value that is more than 1.5 times the IQR above the 75th percentile or below the 25th percentile is considered an outlier. This resulted in the exclusion of 12 cases from the original sample of 196 cases.

### Methods

General clinical data of the patients were collected; the primary output variables were CK-MB/CK ratio and NLR. Peripheral fasting venous blood samples were collected using vacuum tubes containing dipotassium-ethylene diamine tetra-acetic acid and heparin as anticoagulants. After blood collection, the anticoagulant tubes were gently mixed to prevent clot formation and bubble generation. The CK and CK-MB levels were measured using a Hitachi 7600 fully automatic biochemical analyser, and the CK index (CK index = CK-MB/CK) was calculated [7]. The SYSMEX IE2100 fully automatic blood cell analyser was used to measure the white blood cells, NEUT, LYM counts, etc., and the NLR was calculated (NLR = NEUT/LYM). Original matching reagents were used, with internal quality evaluations performed using these before blood sample testing was conducted.

### The diagnosis of acute myocardial infarction

Identifying patients with AMI involves a combination of clinical assessment, diagnostic tests and medical history. Patients with AMI often present with typical symptoms, such as chest pain or discomfort, which may radiate to the arm, neck, jaw, shoulder or back. Other symptoms can include shortness of breath, nausea, light-headedness and sweating. In addition, electrocardiography is a crucial diagnostic tool for identifying AMI in patients; it can show characteristic changes, such as ST-segment elevation or ST-segment depression. Furthermore, coronary angiography can be performed in cases where revascularisation procedures, such as percutaneous coronary intervention or coronary artery bypass grafting, are considered, as it provides direct visualisation of coronary artery blockages.

### Data analysis

Data analyses were performed using SPSS v.22.0 (IBM Statistical Software™, IBM Corp., Armonk, NY, USA). The hypothesis testing was as follows:

- Null hypothesis (H0): There is no significant association between CK-MB/CK, NLR and red blood cell distribution width and the occurrence of AMI in the studied population.
- Alternative hypothesis (H1): There is a significant association between CK-MB/CK, NLR and red blood cell distribution width and the occurrence of AMI in the studied population.

Data pre-processing mainly included identifying and handling missing data by mean/median imputation, either removing the corresponding records or filling in the missing values. In addition, outliers that may have distorted the analysis were identified. Inconsistencies or errors in the data were corrected and the numerical features were standardised or normalised to ensure they had a similar scale. Statistical methods, such as the interquartile range or *z*-score, were employed to identify values that fell significantly outside the expected range.

### Statistical analysis

Statistical analyses were performed using SPSS v.22.0 (SPSS, Inc., Chicago, IL, USA). All measurement data were tested for normality using the Kolmogorov–Smirnov test and presented as the mean ± standard deviation (x̅ ± s). Independent sample *t*-tests were used for comparing data between groups. Multivariate logistic regression analysis was used to identify risk factors for AMI occurrence based on the differences between the groups using model relationships between variables. Receiver operating characteristic (ROC) curve analysis was employed to evaluate the predictive performance. A significance level of *p* < 0.05 denoted statistical significance.

## Results

### Comparison of the serum level results between the groups

As Table 1 shows, the serum levels of CK, CK-MB, CK index, NEUT and NLR in the AMI group were significantly higher compared with those in the control group (*p* < 0.05). However, the LYM serum levels were significantly lower in patients with AMI compared with the levels in the control group (*p* < 0.05).

**Table 1:** Comparison of CK, CK-MB, CK index, LYU, NEUT, and NLR levels between two groups

Group	n	CK(mmol/L)	CKMB(mmol/L)	CK index	LYU(×10^9^/L)	NEUT(×10^9^/L)	NLR
AMI Group	196	304.42 ± 297.61	28.07 ± 25.49	0.11 ± 0.08	1.37 ± 0.89	7.14 ± 3.82	7.37 ± 6.63
Control Group	144	114.65 ± 65.33	8.64 ± 2.83	0.09 ± 0.04	2.03 ± 0.64	3.42 ± 1.00	1.79 ± 0.62
t		6.57	6.13	3.29	-7.537	11.39	10.01
P		< 0.001	< 0.001	0.001	0.001	< 0.001	< 0.001

Note: CK-MB: Creatine kinase-MB; LYU: Lymphocyte; NEUT: Neutrophils; NLR: Neutrophils to lymphocytes ratio

### Multivariable logistic regression analysis of the factors influencing the occurrence of acute myocardial infarction

The multivariate logistic regression analysis of CK, CK-MB, CK index, LYM, NEUT and NLR revealed that elevated CK-MB and NLR were independent risk factors for AMI occurrence (Table 2).

**Table 2:** Multivariable logistic regression analysis of factors influencing AMI patients

Influencing Factors	B-value	SE-value	Wald	*P*-value	OR (95%Cl)
CK	0.004	0.005	0.653	0.419	1.004 (0.994 ~ 1.014)
CKMB	0.221	0.085	6.804	0.009	1.248 (1.057 ~ 1.473)
CK index	-1.994	7.235	0.072	0.788	0.143 (0.009 ~ 9.862)
LYU	1.226	0.596	4.233	0.060	3.409 (1.060 ~ 10.963)
NEUT	-0.437	0.334	1.709	0.191	0.646 (0.336 ~ 1.244)
NLR	2.392	0.626	14.603	0.000	10.940 (3.209 ~ 37.302)

Note: CK-MB: Creatine kinase-MB; LYU: Lymphocyte; NEUT: Neutrophils; NLR: Neutrophils to lymphocytes ratio

### The receiver operating characteristic curve analysis of the CK index and NLR for predicting acute myocardial infarction

**Fig. 1:**
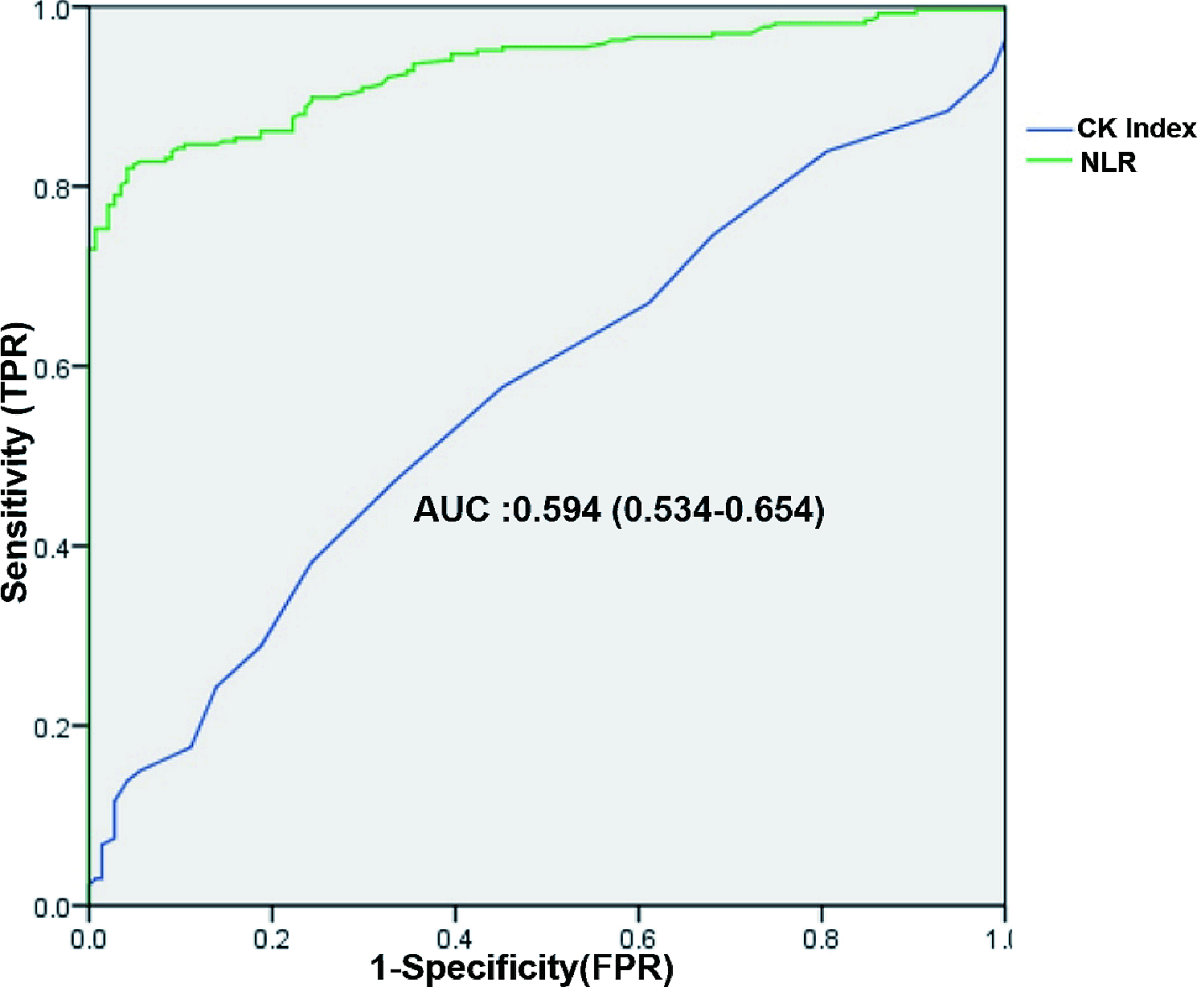
ROC Curve Analysis of NLR and CK Index. False Positive Rate (FPR), True Positive Rate (TPR); Area Under the Curve (AUC)

The ROC curve analysis showed that NLR’s area under the curve (AUC) for predicting AMI was 0.917 (95% CI: 0.888–0.947, *p* < 0.001), with a sensitivity of 83.7% and specificity of 81.2%. The AUC for the CK index was 0.594 (95% CI: 0.534–0.654, *p* = 0.003), with a sensitivity of 60.2% and specificity of 54.9% (Fig. 1).

## Discussion

Acute myocardial infarction is a common cardiovascular emergency disease caused by acute obstruction of the coronary artery leading to local myocardial ischemic necrosis. Inflammation and oxidative stress are related to its pathogenesis, with atherosclerosis being the pathological basis for the development of the disease. Therefore, the role of inflammatory biomarkers in the occurrence of AMI has received extensive attention from local and international scholars [8–10]. The NLR is an inflammatory marker that combines two subtypes of white blood cells, the NEUTs and LYMs, and has been demonstrated to be associated with the occurrence and prognosis of cardiovascular and cerebrovascular diseases [11–13]. 

Neutrophils can aggravate endothelial cell injury and the damaged endothelial cells can induce NEUT adhesion and aggregation through the expression of selectins and chemokines. The interaction between the NEUTs and the basement membrane increases the permeability of the endothelium [14], facilitating the aggregation of monocytes/macrophages, promoting the transformation of macrophages into foam cells, and forming plaques [15]. When atherosclerosis affects the smooth muscle cell layer of the media, activated smooth muscle cells attract the NEUTs to promote plaque rupture and thrombus formation [16]. 

Lymphocytes are important cells involved in specific immunity and have a regulatory role in inflammatory responses, protecting arterial vessels by inhibiting inflammatory responses during the occurrence and development of atherosclerosis [17]. In this study, the NLR in patients with AMI was significantly higher compared with the control group (*p* < 0.05). In addition, multivariate logistic regression analysis showed that NLR elevation was a factor influencing the occurrence of AMI in patients (*p* < 0.05). The ROC curve analysis revealed an AUC of 0.917 for the NLR predicting AMI, with a sensitivity and specificity of 83.7% and 81.2%, respectively. These findings suggest that NLR elevation is associated with the occurrence of AMI.

Creatine kinase mainly exists in the myocardium and skeletal muscles of the human body, including three types of isoenzymes: muscle-type (CK-MM), brain-type (CK-BB) and heart-type (CK-MB), which mainly exists in the myocardium and is a sensitive indicator for early detection of myocardial injury in clinical practice. In addition, the CK-MB level is often used in conjunction with electrocardiography for the clinical diagnosis of patients with chest pain [18]. However, as the understanding of different systemic diseases deepens, it has been found that malignancies, severe liver diseases, skeletal muscle loss, severe trauma, and other conditions may cause a false elevation of CK-MB. In clinical practice, a CK-MB value exceeding twice the upper limit of normal is often indicative of a higher possibility of acute myocardial injury, but this empirical criterion may lead to clinical misdiagnosis. Subsequently, some scholars have proposed that using CK-MB/CK as an index for the diagnosis and differential diagnosis of myocardial injury has advantages over CK-MB alone [19, 20]. In this study, the CK index in patients with AMI was significantly higher compared with the control group (*p* < 0.05). The ROC curve analysis revealed an AUC of 0.594 for the CK index, with a sensitivity of 60.2% and a specificity of 54.9%; this suggests that CK-MB/CK has a moderate diagnostic efficacy.

In conclusion, the CK index and NLR have a predictive value for the diagnosis of AMI, with the NLR being superior to the CK index. However, due to the small sample size of this study, the results may be biased, limiting its clinical application. Therefore, further validation is needed through larger-scale clinical studies.

## Data Availability

All data generated or analysed during this study are included in this article. Further enquiries can be directed to the corresponding author.

## Abbreviations

WBC: White blood cells
NEUT: Neutrophils
LYU: Lymphocyte
AMI: Acute myocardial infarction
CK-MB: Creatine kinase-MB
NLR: Neutrophils to lymphocytes ratio
ROC: Receiver operating characteristic curve
